# Primary Solitary Intra-thyroid Myofibroma in a Pediatric Patient

**DOI:** 10.7759/cureus.99847

**Published:** 2025-12-22

**Authors:** Lillie Jensen, Gleidson Silva, Tamarah Westmoreland, Lili Miles

**Affiliations:** 1 Pathology, University of Central Florida College of Medicine, Orlando, USA; 2 Radiology, Nemours Children's Hospital, Orlando, USA; 3 Pediatric Surgery, Nemours Children's Hospital, Orlando, USA; 4 Pathology, Nemours Children's Hospital, Orlando, USA

**Keywords:** benign thyroid lesions, fine-needle aspiration (fna), myo-fibroblastic tumour, myofibroma, pediatric solid tumors, pediatric thyroid lesions, soft tissue pathology, solitary infantile myofibroma, thyroid mass, tissue biopsy

## Abstract

Myofibromas are benign soft tissue tumors, mostly seen in young children, and characterized histologically by fibroblasts/myofibroblasts arranged in fascicles. Clinical presentation of this tumor is highly variable, based on the location and extent of organ involvement. Myofibromas most commonly involve skin and subcutaneous tissue, though they may appear in any location and may be multiple. Nearly all thyroid lesions in children are derived from thyroid follicular cells or C-cells. Solitary myofibroma occurring in the thyroid is exceptionally rare. This case report describes a three-year-old male presenting with a right neck mass. Fine needle aspiration (FNA) revealed spindle cells without evidence of malignancy. Resection of the right thyroid lobe and isthmus revealed a myofibroma, which was completely excised. The patient remained healthy without recurrence two years after the surgery. This case highlights that a solitary myofibroma can present as an intra-thyroid lesion in a pediatric patient. Histological evaluation is critical to establish the diagnosis of myofibroma, and ancillary studies may be necessary to differentiate it from other potential differential diagnoses of spindle cell morphology.

## Introduction

Myofibromas are benign soft tissue tumors, characterized histologically by spindle cells (myofibroblasts) arranged in fascicles. The incidence of myofibromas is approximately one in 150,000 - 400,000 [1]. These mesenchymal tumors most commonly affect young children and represent approximately 12% of all pediatric soft tissue tumors [2]. Myofibromas present with considerable clinical variability and are classified into subtypes based on the number and extent of lesions: “solitary,” multicentric “myofibromatosis,” or widespread “generalized myofibromatosis” subtypes [3]. Primary solitary myofibroma involving solid organs is uncommon, and solitary myofibroma occurring in the thyroid is exceptionally rare, with less than five cases reported in the literature. Common solid lesions of the thyroid are benign, identified as either goitrous (hyperplastic) nodules or thyroid adenomas. An estimated 20-25% of all pediatric thyroid nodules are malignant, primarily composed of papillary thyroid carcinoma, followed by thyroid follicular carcinoma and medullary carcinoma [4]. 

Grossly, myofibromas appear as well-circumscribed nodular lesions less than 10 cm in diameter, though deeper myofibromas may have less well-defined margins. These masses are firm with a tan-white color and are found with or without hemorrhage or calcifications. Key histologic features include the presence of elongated spindle cells (fibroblasts/myofibroblasts) in whorls, arranged in short fascicles and sheets. Immunohistochemical analysis of myofibroblasts exhibits positive staining with smooth muscle actin (SMA) and variable staining with desmin. These spindle cells stain negative for CD34, S-100, and epithelial or vascular markers [5]. Myofibromas generally have a very good prognosis, with cutaneous manifestations often regressing spontaneously. Predictors of poor prognosis include the presence of multiple lesions or visceral involvement [6]. When located within solid organs, myofibromas are frequently treated with surgical resection, which is both diagnostic and therapeutic [7,8].

## Case presentation

**Figure 1:**
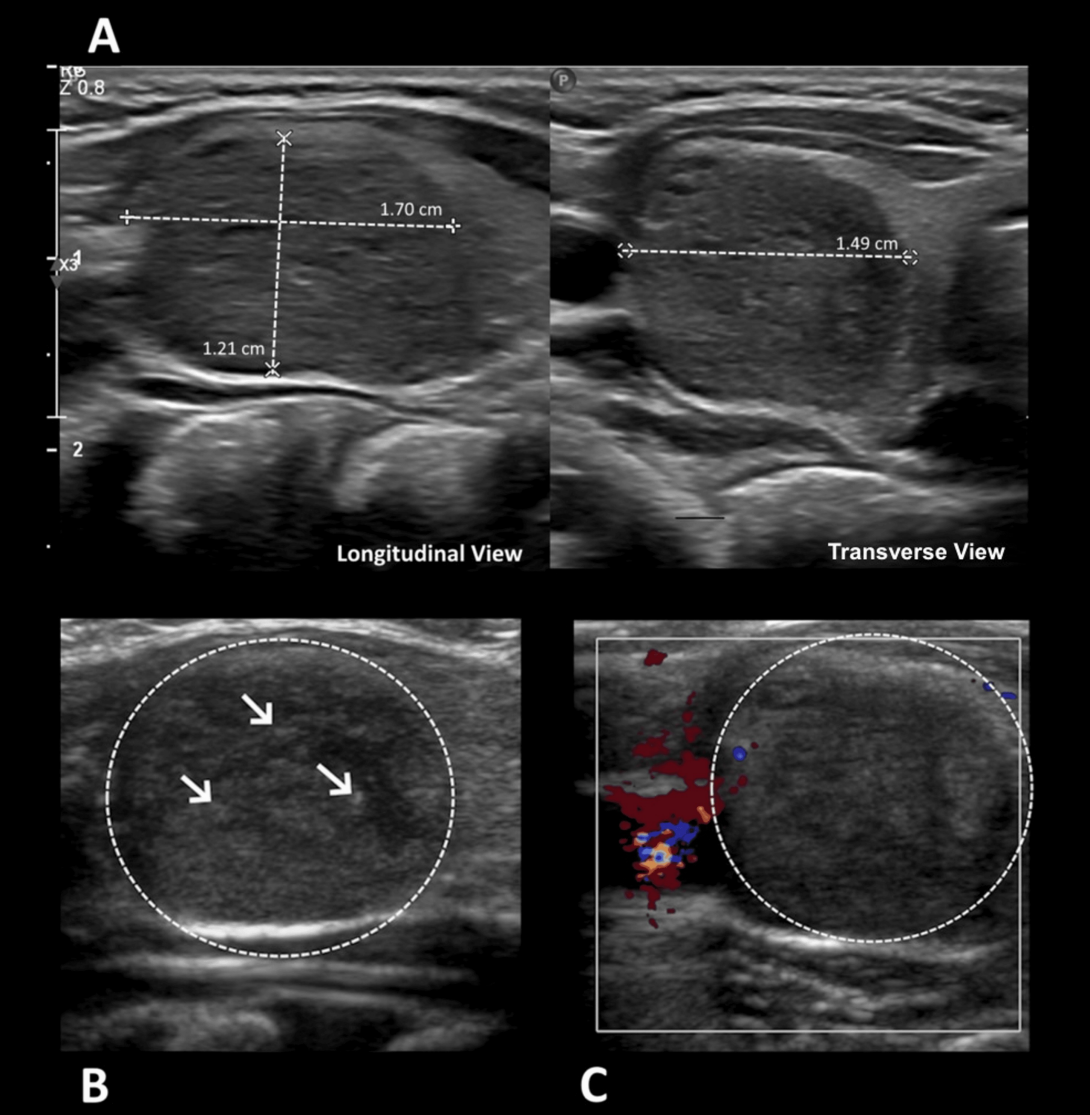
Ultrasound findings A: grayscale ultrasound shows a nonspecific, well-defined, hypoechoic, heterogeneous, solid, ovoid nodule with smooth margins (between cursors) that was found within the right thyroid lobe; B: there are multiple punctate echogenic foci (arrows), likely microcalcifications within the nodule (circled); C: color Doppler ultrasound demonstrates minimal vascularity of the nodule (circled). Overall, these findings are nonspecific and are seen in multiple entities occurring in the thyroid.

The patient is a previously healthy three-year-old boy with no significant past medical history. The parents noticed a right neck mass that gradually increased in size over a week. They reported to the child’s primary care provider that he had increased snoring when supine, though they denied any additional symptoms associated with swallowing or breathing. The provider ordered an ultrasound, which detected a 1.5 cm right thyroid nodule (Figure 1).

**Figure 2:**
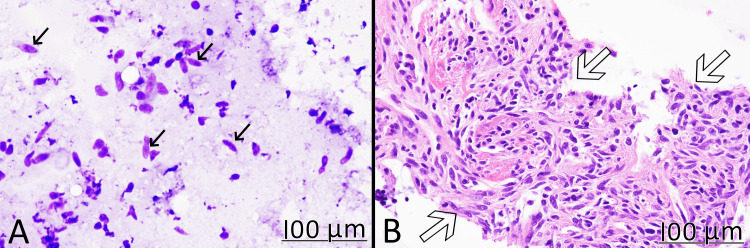
FNA specimen A: Wright-Giemsa staining showed scattered spindle cells (black arrows) without significant cytologic atypia (x40); B: cell block confirmed a spindle cell tumor (hollow arrows) without cytological pleomorphism or other indication of malignancy (H&E x40). FNA: fine needle aspiration

The patient was euthyroid and was otherwise normal for his age. Fine needle aspiration (FNA) was performed three weeks later, which revealed spindle cells without pleomorphism. The diagnosis on FNA was a spindle cell tumor without morphologic evidence of malignancy (Figure 2). While the cytology was benign, sampling bias cannot be completely ruled out. Surgical removal was recommended since the tumor was confined to one lobe. Examination of the entire tumor can make a definitive diagnosis and completely rule out malignancy. 

**Figure 3:**
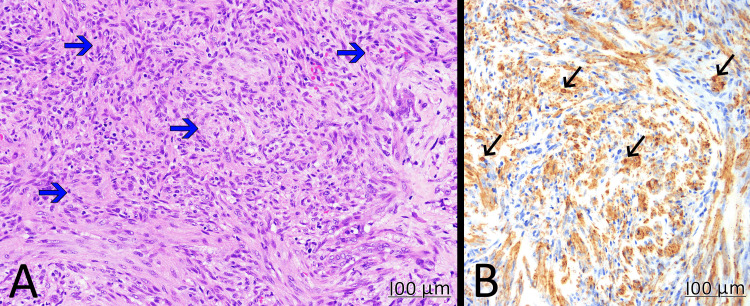
Resection specimen A: H&E stain (x40) showed spindle cells arranging in fascicles (blue arrows) with variable cellularity, but without pleomorphism; B: spindle cells (black arrows) were diffusely positive for SMA (immunohistochemistry stain x40). SMA: smooth muscle actin

A right thyroid lobectomy, including isthmectomy, was subsequently performed. Gross examination revealed a 1.5 x 1.5 x 1.2 cm solid nodule with a homogenous, firm, white surface without hemorrhage or necrosis, contained entirely within the submitted right thyroid lobe. The background thyroid tissue was grossly normal. Histologically, the tumor was composed of spindle cells forming a vague, fascicular pattern. There was no pleomorphism. The mitoses were approximately one figure per 10 high-power fields. The spindle cells exhibited positive immunohistochemical staining for SMA and negative staining for CK7, SS18-SSX, CD34, beta-catenin, pan cytokeratin, ALK, BRAF, desmin, and myogenin. These findings are consistent with a diagnosis of myofibroma (Figure 3). Surgical resection margins were negative for tumor.

Due to the rarity of primary thyroid myofibromas, an external second opinion was obtained to confirm the diagnosis. Considering the possibility of multicentric or generalized myofibromatosis subtypes, which are associated with poor prognosis and significant morbidity, a full body screen was performed via physical examination, revealing no other lesions. Post-surgically, the patient remained in good health with no recurrence for two years up to date.

## Discussion

Most intra-thyroid masses in pediatric patients are derived from thyroid follicular cells or parafollicular C-cells. Lesions of these origins commonly include nodular goiters and adenomas, or, less commonly, papillary, follicular, or medullary carcinoma. When a thyroid mass is suspected, ultrasound is the imaging modality of choice and is used to identify benign versus malignant features. Depending on findings, FNA is the first choice for histological evaluation. Based on the cytologic and/or molecular results, the management ranges from observation to partial or total thyroidectomy [9]. Primary intrathyroidal spindle cell tumors are exceedingly rare. 

Myofibromas are benign solitary tumors composed of fibroblasts and myofibroblasts that most commonly occur in the soft tissue of the head and neck in young children under two years old [10]. Involvement of solid organs is more likely associated with widespread and severe disease, referred to in the literature as generalized myofibromatosis [3,10]. Of the solitary form, only approximately five percent arise within a solid organ. The largest clinicopathological review to date included 97 cases of myofibromas, which included four patients with internal organ involvement (three colon, one small bowel) [10]. Intra-thyroid myofibromas are exceptionally rare, with only two pediatric cases found in the literature by us. In the first case, a euthyroid five-year-old female presented with a large (4 x 2.5 x 2.5 cm), solitary, firm mass on the anterior neck. Ultrasound revealed a lesion in the right lobe of the thyroid, and a subsequent biopsy revealed it to be a solitary myofibroma. Simple surgical tumor excision followed, and the patient had no signs of recurrence two years following the procedure [8]. In the second case, a male neonate described an extensive neck mass comprised of both the thyroid gland and the left submandibular gland, causing airway obstruction. Biopsy of the mass identified a solitary myofibroma, and the tumor was determined to be inoperable [7]. Table 1 summarizes the clinical and histologic features of intra-thyroid myofibromas in the literature compared to our index case. 

**Table 1:** Summary Comparison of the index case with the clinical, histologic, management, and follow-up results of two similar reports in the literature.

Patient	Clinical presentation	Sites involved	Imaging findings	Histological diagnosis	Treatment	Follow-up results
3-year-old male (index case)	1-week history of right neck mass and snoring	Right thyroid	Ultrasound: well-defined, hypoechoic solid nodule with echogenic foci	Needle biopsy: spindle cell tumor excisional biopsy (myofibroma)	Right thyroid lobectomy	Disease-free for 2 years
5-year-old female [7]	1-day history of frontal neck swelling	Right thyroid	Ultrasound: inhomogeneous, low-echogenic tumorous mass without any calcifications	Excisional biopsy: myofibroma	Simple tumor excision	Disease-free for 2 years
1-day-old male [6]	Airway compromise	Extensive; see imaging findings	MRI: large abnormality extending from the oropharynx to the thoracic inlet, with involvement of the thyroid and left submandibular gland	Transbronchial biopsy: myofibroma	Tracheostomy, chemotherapy	Stable in size for 3 months

Our patient represents the third case of intra-thyroid myofibroma. Due to the nonspecific appearance of these lesions on physical examinations and similar imaging findings compared to other intra-thyroid lesions, tissue examination remains the gold standard for diagnosis. Patients diagnosed with myofibroma must receive full-body screening to exclude generalized disease, though serial examinations are generally unnecessary due to low rates of recurrence and excellent prognosis following removal. In a systematic review spanning 50 cases of myofibroma treated with surgical removal, tumor recurrence occurred in just seven cases, or 15% (at a median follow-up interval of 45.5 months), despite an overall high rate (78%) of positive margins. The reported recurrences were local and nonaggressive in nature, with no cases involving distant metastases or death. The findings of that literature review highlight the overall benign and indolent nature of solitary myofibroma, and while surgical excision is the most frequently recommended approach, watchful waiting may be feasible in some scenarios [11].

## Conclusions

This report underscores the importance of tissue examination of all thyroid surgical specimens, an example and reminder that rare lesions such as solitary myofibroma may present unexpectedly, especially in pediatric patients. It is necessary to identify intra-thyroid lesions not derived from thyroid follicular epithelial cells or C-cells to facilitate a definitive diagnosis and inform the appropriate level of treatment. This case conveys that myofibroma must be part of the broad differential for a solitary mass in solid organs such as the thyroid. The reporting of a solitary myofibroma in this exceedingly rare and unusual location contributes to the literature in awareness and description of the clinical course following the resection of this tumor.
